# Antioxidant activity of cerium dioxide nanoparticles and nanorods in scavenging hydroxyl radicals†

**DOI:** 10.1039/c9ra00642g

**Published:** 2019-04-09

**Authors:** Alexander Filippi, Fobang Liu, Jake Wilson, Steven Lelieveld, Karsten Korschelt, Ting Wang, Yueshe Wang, Tobias Reich, Ulrich Pöschl, Wolfgang Tremel, Haijie Tong

**Affiliations:** Multiphase Chemistry Department, Max Planck Institute for Chemistry Mainz 55128 Germany h.tong@mpic.de; School of Chemical and Biomolecular Engineering, Georgia Institute of Technology Atlanta Georgia 30332 USA; Institute for Inorganic Chemistry and Analytical Chemistry, Johannes Gutenberg University Mainz Mainz 55128 Germany; State Key Laboratory of Multiphase Flow in Power Engineering, Xi'an Jiaotong University Xi'an 710049 China; Institute of Nuclear Chemistry, Johannes Gutenberg University Mainz Mainz 55099 Germany

## Abstract

Cerium oxide nanoparticles (CeNPs) have been shown to exhibit antioxidant capabilities, but their efficiency in scavenging reactive oxygen species (ROS) and the underlying mechanisms are not yet well understood. In this study, cerium dioxide nanoparticles (CeNPs) and nanorods (CeNRs) were found to exhibit much stronger scavenging activity than ·OH generation in phosphate buffered saline (PBS) and surrogate lung fluid (SLF). The larger surface area and higher defect density of CeNRs may lead to higher ·OH scavenging activity than for CeNPs. These insights are important to understand the redox activity of cerium nanomaterials and provide clues to the role of CeNPs in biological and environmental processes.

## Abstract 2

Cerium dioxide nanoparticles and nanorods were found to exhibit much stronger scavenging activity than ·OH generation in quasi-physiological conditions.

## Graphical abstract

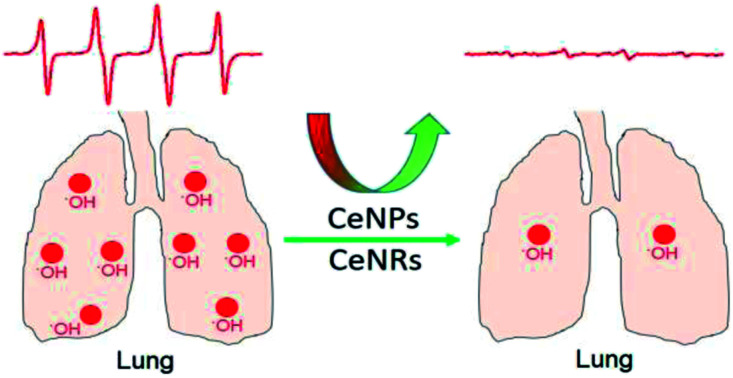

Reactive oxygen species (ROS) generally describe reduction products of oxygen molecules, including H_2_O_2_ and hydroxyl radicals (·OH).^1^ ROS play a central role in biological processes exerting both beneficial and adverse health effects.^2^ Several studies have looked into the redox balance between ROS and antioxidants^3^ as well as the underlying mechanisms.^4^ Among all ROS, ·OH is considered as one of the most reactive species; it can attack biomolecules and cause irreversible damage.^5^ Thus, experimental quantification and abiotic regulation of ·OH under physiologically relevant conditions is an important yet challenging task.

In the last decade, cerium dioxide nanoparticles (CeNPs) have drawn much attention due to their redox properties^6^ and potential therapeutic applications (such as treating cardiac ischemia).^7–9^ Efforts have been made to explore the potential use of CeNPs as medicine.^7,10,11^ The ability of CeNPs in switching the oxidation state of Ce^3+^ and Ce^4+^ makes it a good candidate to mediate ROS.^6,12^ Direct scavenging of ·OH (process ① in Scheme 1), NO·, and OONO^−^ by CeNPs have been investigated.^13–16^ Moreover, previous studies indicated that CeNPs have catalase- and superoxide dismutase (SOD)-like effects (processes ③ and ⑤ in Scheme 1).^17,18^ Both effects are closely correlated with the Ce^3+^ and Ce^4+^ surface concentrations, pH, H_2_O_2_ and chelating ligand concentrations.^19–23^

**Scheme 1:**
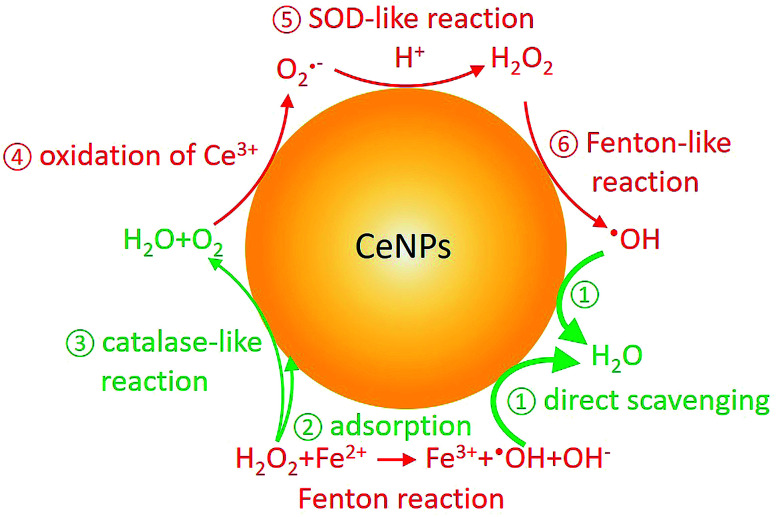
Fenton reaction and reactive oxygen chemistry of CeNPs. Red and green colors indicate ROS formation and scavenging processes, respectively.

In contrast to research about the antioxidant activity of CeNPs, inhalable CeNPs have been detected in ambient air and concerns have been raised about their potential adverse health effect.^24,25^ Besides this, additional studies suggested that CeNPs can induce oxidative stress, inflammatory signaling response, and cell death upon generating ROS (processes ④–⑥ in Scheme 1) or ROS-messengers.^26–30^ Given the controversies about the beneficial and toxic effects of CeNPs, it is necessary to distinguish the anti- and prooxidant activities of CeNPs under physiologically relevant conditions.^31^ In this study, we compared the ·OH formation and scavenging ability of commercial CeNPs (*∅* 25 and 50 nm) and homemade cerium nanorods (CeNRs) with different physicochemical properties in phosphate buffered saline (PBS) buffer, antioxidant solutions, and a surrogate lung fluid (SLF). The SLF was used to mimic the key interface between human respiratory tract and inhaled air.

Fig. 1 shows the size, morphology, surface composition, and mass normalized surface area of CeNPs and cerium dioxide nanorods (CeNRs). More information about the applied techniques, sample handling and instrument settings is compiled in Sections S1–S5.†Fig. 1A and B indicate that CeNPs (*∅* 50 nm) and CeNPs (*∅* 25 nm) have a heterogeneous size distribution with average diameters of <50 nm and <25 nm respectively. Moreover, samples of these commercial CeNPs contain predominantly cubic NPs. In contrast, the morphology of CeNRs (Fig. 1C) is more uniform with a length of ∼100 nm. Details about the CeNRs can be found from our previous study.^32^ In addition to the detection of size and morphology, the specific surface areas of the cerium nanoparticles were determined to be 24.8 ± 0.4 m^2^ g^−1^ (*∅* 50 nm CeNPs) (Fig. 1D), 39.2 ± 0.7 m^2^ g^−1^ (*∅* 25 nm CeNPs) (Fig. 1E), and 106.5 ± 2.4 m^2^ g^−1^ (CeNRs) (Fig. 1F). Moreover, the similar Ce 3d XPS spectra of CeNPs (*∅* 50 nm) (Fig. 1G), CeNPs (*∅* 25 nm) (Fig. 1H), and CeNRs (Fig. 1I) indicate that the distribution of the cerium surface oxidation states (Ce^3+^ and Ce^4+^) on these NPs are quite similar. The six most prominent peaks of these spectra are attributable to Ce^4+^ ions.^33^ This indicates that Ce^4+^ was the dominant cerium species in all three samples. The peak fittings (dashed lines) in panels G, H and I are based on the method by Maslakov *et al.*.^33^ The fitting based deconvolution of Ce 3d XPS spectra indicates that the concentration of surface Ce^3+^ in all these samples is <3%. Such a low abundance of surface Ce^3+^ is also supported by the absence of a shoulder peak of Ce 4f electrons at ∼1.1 eV in the XPS valence band spectrum of the CeNRs samples (Fig. S3†). Furthermore, the deconvolution of the XPS spectrum of the O 1s region of the NPs (Fig. S2 and Table S3†) indicates that the CeNRs surface contains a much higher concentration of hydroxide than CeNPs. This may correlate with the synthesis method of CeNRs using NaOH as reagent^34^ and may play a role in the higher ·OH scavenging activity of CeNRs. These differences in chemical composition, morphology, and surface area between CeNPs and CeNRs may result in variations of their redox activity.

**Fig. 1:**
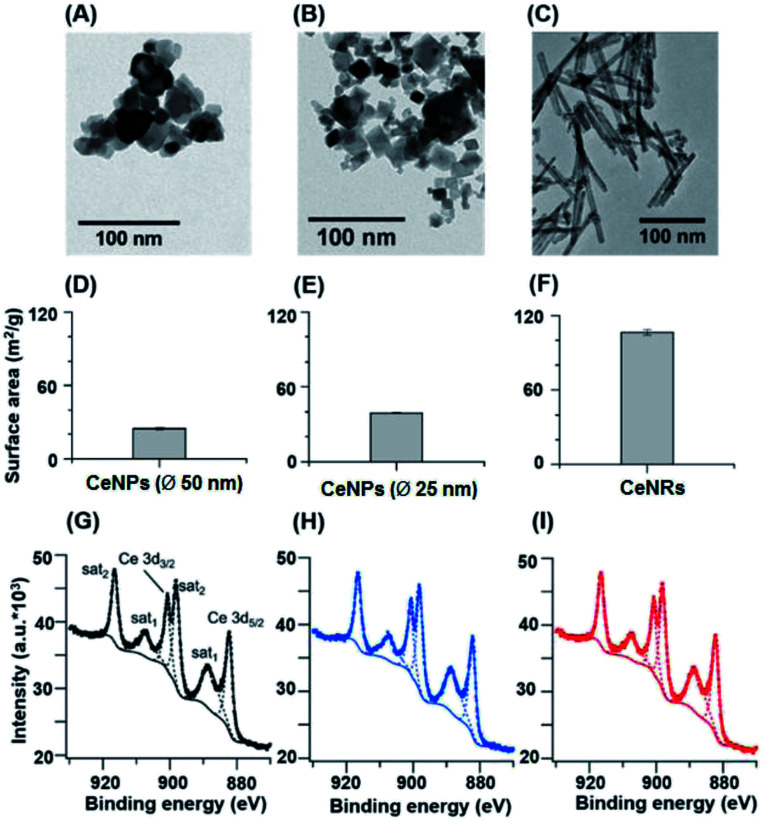
Physicochemical characteristics of CeNPs (*∅* 50 nm) (A, D, and G), CeNPs (*∅* 25 nm) (B, E, and H), and CeNRs (C, F, and I). (A–C) TEM images. (D–F) Surface areas determined by BET. (G–I) Ce 3d XPS spectra. The error bars in panels (D–F) represent standard deviations based on three replicates. The dashed lines in panels (G–I) are fitting curves.

Fig. 2 shows the trapping mechanism of 5-*tert*-butoxycarbonyl-5-methyl-1-pyrroline-*N*-oxide (BMPO, panel A) and EPR spectra of aqueous mixtures of Fe^2+^, H_2_O_2_, SLF, and CeNPs (panel B). Fig. 2A shows that BMPO can react with OH radicals and form a BMPO–OH radical adduct. In this way, short lifetime radicals can be probed and characterized by electron paramagnetic resonance (EPR) spectroscopy (EMXplus10/12, Bruker, Germany, see details in Section S5 and Table S4†). The grey dashed lines in panel B indicate the characteristic hyperfine splitting of BMPO–OH, in agreement with previous assignment.^35^ The peak intensities of the spectra in Fig. 2B decrease in the order A (Fe^2+^ + H_2_O_2_) > B (Fe^2+^ + H_2_O_2_ + CeNPs) > C (Fe^2+^ + H_2_O_2_ + SLF) > D (Fe^2+^ + H_2_O_2_ + CeNPs + SLF). This implies that the amount of ·OH decreases accordingly. Based on the spin-counting method,^32^ we quantified the concentration of BMPO–OH in these solutions. The results are shown in Fig. 3 and Tables S5–S7.†

**Fig. 2:**
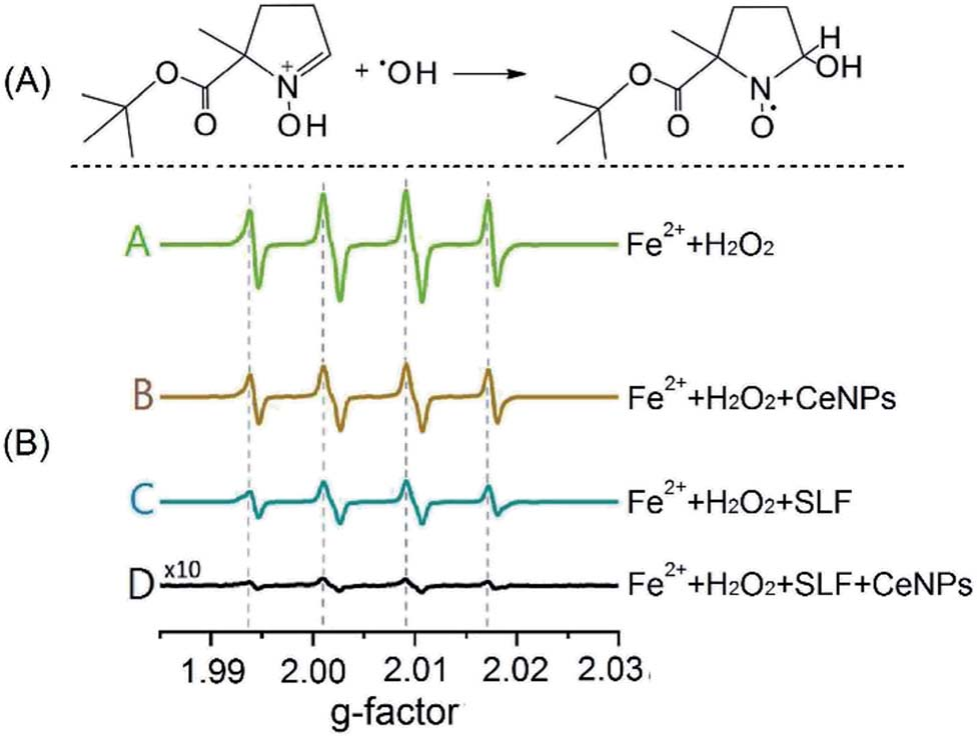
(A) Reaction mechanism of the spin-trapping agent BMPO with a hydroxyl radical. (B) EPR spectra of the BMPO-radical adduct in different aqueous mixtures. The four peaks (dotted lines) are characteristic of BMPO–OH adducts. The concentrations of Fe^2+^, H_2_O_2_, and CeNPs (*∅* 50 nm) are 1 mM, 10 mM, and 10 mg mL^−1^, respectively.

**Fig. 3:**
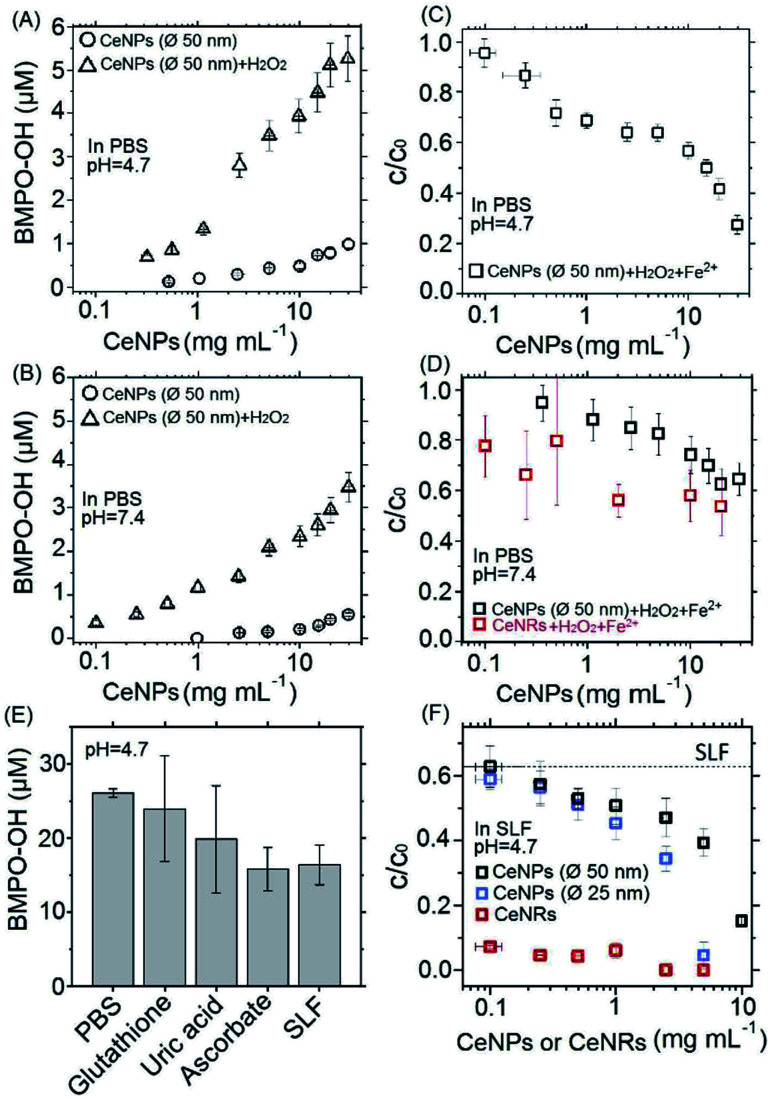
Concentrations (A, B, and E) or remaining fractions (C, D, and F) of BMPO–OH in different aqueous mixtures. (A) and (B) Concentrations of BMPO–OH formed by pure CeNPs (*∅* 50 nm) (○) or their mixtures within H_2_O_2_ (Δ) in pH = 4.7 (A) and 7.4 (B) PBS. (C) and (D) Remaining fraction (*c*/*c*_0_) of BMPO–OH without (*c*_0_) and with (*c*) mixing CeNPs (*∅* 50 nm) or CeNRs (□) with 1 mM Fe^2+^ and 10 mM H_2_O_2_ in pH = 4.7 (C) and 7.4 (D) PBS. (E) Concentration of BMPO–OH formed by Fenton reactions in neutral PBS, antioxidant solutions, and SLF. (F) *c*/*c*_0_ of BMPO–OH with and without mixing CeNPs (*∅* 50 nm) (□), CeNPs (*∅* 25 nm) (□), and CeNRs (□) with 1 mM Fe^2+^ and 10 mM H_2_O_2_ in pH = 4.7 SLF. The values of *c*_0_ in panels B, D and F are ∼53, ∼17, and ∼26 μM. The *x*-axis errors in panels A, B, C, D, and F represent uncertainties from weighing and pipetting. All the *y*-errors represent standard deviation of more than three replicates.

Fig. 3A and B show the positive correlation of ·OH yields of CeNPs (*∅* 50 nm) without (black circles) and with the addition of H_2_O_2_ (black triangles) under different CeNPs (*∅* 50 nm) loading conditions. In the absence of H_2_O_2_, 0.1–30 mg mL^−1^ CeNPs (*∅* 50 nm) can generate 0–0.8 and 0–0.5 μM ·OH in pH = 4.7 (Fig. 3A) and 7.4 (Fig. 3B) PBS, respectively. The generation of ·OH by pure CeNPs (*∅* 50 nm) in acidic PBS is consistent with previous hypothesis that acid can catalyze the ·OH formation by CeNPs.^36^ In contrast to pure CeNPs (*∅* 50 nm), mixtures of 0.1–30 mg mL^−1^ CeNPs (*∅* 50 nm) with 10 mM of H_2_O_2_ can generate 0–5 (pH = 4.7) and 0–3 μM (pH = 7.4) ·OH, which also shows a positive correlation with the loading of CeNPs (*∅* 50 nm) as shown in Fig. 3A and B. These hydroxyl radicals may be formed through Fenton-like reactions initiated by CeNPs:^37^ H_2_O_2_ + Ce^3+^ → Ce^4+^ + ·OH + OH^−^.

To evaluate the ·OH scavenging activity of CeNPs in aqueous solution, we measured the ·OH yield by mixtures of CeNPs (*∅* 50 nm) or CeNRs, Fe^2+^, and H_2_O_2_ in acidic and neutral PBS. Fig. 3C and D show that the ·OH concentration decreased with increasing CeNPs or CeNRs loading, characterized by the decreasing remaining OH radical concentration. In the absence of CeNPs, Fenton reactions of 1 mM Fe^2+^ and 10 mM H_2_O_2_ generated ∼53 and ∼17 μM ·OH in pH = 4.7 (Fig. 3C) and 7.4 (Fig. 3D) PBS. At 30 mg mL^−1^ CeNPs (*∅* 50 nm), concentration of ·OH decreased to 15 μM (pH = 4.7) and 11 μM (pH = 7.4), respectively (Tables S5 and S6†). In contrast to CeNPs, CeNRs exhibited higher ·OH scavenging efficiency, with 20–50% of ·OH to be scavenged by 0.1–20 mg mL^−1^ CeNRs. This implies that the scavenging activity of CeNPs (*∅* 50 nm) is more pronounced under acidic conditions. The decrease of ·OH concentration may be induced by the following processes: first, CeNPs (*∅* 50 nm) or CeNRs could scavenge ·OH directly (process ① in Scheme 1).^13^ Second, the adsorption of H_2_O_2_ on CeNPs (*∅* 50 nm) or CeNRs surfaces (like process ② in Scheme 1) may decrease the available H_2_O_2_ concentration.^38^ In this case, due to the lower availability of the H_2_O_2_ precursor, the amount of ·OH formed by Fenton reactions will decrease. Third, the surface-bound H_2_O_2_ can be decomposed *via* catalase-like reactions (process ③ in Scheme 1).^21^ This process will form H_2_O and O_2_ rather than ·OH. Beyond these two pathways, iron ion-initiated redox processes may also influence the measured ·OH concentrations. For instance, it has been suggested that upon interaction with the surface of CeNPs, Fe^2+^ can enhance the dissolution of Ce^3+^ and cause the formation of 6-line ferrihydrite, which can increase the colloidal stability of the CeNPs.^39^ Such a reaction may alter the redox activity of CeNPs (*∅* 50 nm) or CeNRs.

Recently Baldim *et al.*^38^ measured the H_2_O_2_ surface adsorption potential of CeNPs with different sizes. They found that 5–28 nm diameter CeNPs could adsorb 2–20 H_2_O_2_ molecules nm^−2^, depending on the surface composition of the nanomaterial. We used the adsorption potential from Baldim *et al.* and estimated that only <1% of H_2_O_2_ (∼8 μM) can be adsorbed on the surface of the CeNPs. Therefore, the surface adsorption of H_2_O_2_ by CeNPs cannot fully explain the reduction of ·OH concentration in Fig. 3. Furthermore, Pirmohamed *et al.*^21^ observed a H_2_O_2_ decomposition rate of ∼2.7 nmol min^−1^ through catalase-like reactions. Based on this value, we estimate that a concentration of 0.1 mg mL^−1^ of CeNPs would result in a H_2_O_2_ loss of <2% in our studies. Therefore, we suggest the direct scavenging process (① in Scheme 1), rather than the surface adsorption (② in Scheme 1) and catalase-like (③in Scheme 1) processes to be the dominant reduction pathways of ·OH.

Fig. 3E shows the ·OH scavenging activity of typical epithelial lung fluid antioxidants and a surrogate lung fluid (SLF). Here, 0.1 mM of glutathione, 0.1 mM of uric acid, and 0.2 mM of ascorbate solutions could scavenge ∼8%, ∼14%, and ∼39% of hydroxyl radicals originating from Fenton reactions of 1 mM Fe^2+^ and 10 mM H_2_O_2_ in PBS. The SLF showed a similar activity as 0.2 mM ascorbate, *i.e.* the ·OH scavenging activities of individual antioxidants are not additive and decrease in the order ascorbate > uric acid > glutathione. This trend is consistent with previous findings.^38^

To assess the antioxidant activity of CeNPs under quasi-physiological conditions, we explored the ·OH scavenging activity of CeNPs and CeNRs in SLF. Fig. 3F shows the hydroxyl radical yield by Fenton reactions in SLF as a function of the CeNPs (*∅* 25 and 50 nm) and CeNRs loading. As the loading of CeNPs (*∅* 50 nm) increased from 0.1 to 10 mg mL^−1^, the concentration of ·OH in SLF decreased by 38–85%. Within the same loading range, the CeNPs (*∅* 25 nm) exhibited a similar efficiency. Whereas at higher loadings (1–5 mg mL^−1^), the ·OH scavenging potential of CeNPs (*∅* 25 nm) was 9–55% higher than that of their 50 nm counterparts. In contrast to CeNPs, the CeNRs showed a much higher ·OH scavenging efficiency. Even with a loading as low as 0.1 mg mL^−1^, the CeNRs could reduce 88% of the ·OH. For CeNRs loadings that exceeded 1 mg mL^−1^, no ·OH could be observed. The trend of the ·OH scavenging efficiency according to CeNRs > CeNPs (*∅* 25 nm) >CeNPs (*∅* 50 nm) is in the same order as the surface area of these NPs (Fig. 1D–F). Given the low abundance of Ce^3+^ on fresh CeNPs and CeNRs surface (Fig. 1G–H), we suggest that substantial amount of Ce^3+^ may be formed upon interactions of NPs with water.^13^ The larger surface area of CeNRs may increase the density of Ce^3+^ per unit particle mass and subsequently their ·OH scavenging activity. Previous works showed that CeNRs are prone to expose their (110) facets to reactive species.^34^ These facets were described as reactive “hybrid structures” between the (111) and (100) surfaces of CeNPs. Furthermore, the distinct crystallographic surface structure of CeNRs may act as binding site for reactive species (·OH and H_2_O_2_) exerting peroxidase-like effects. Additionally, Fe^2+^-dependent reactive oxygen chemistry may contribute to the observed ·OH scavenging processes.^39^ Finally, it has been suggested that glutathione could interact with CeNPs and influence the redox couple of Ce^3+^/Ce^4+^.^40^

It is worthy to note that a real physiologic environment is more complicated than SLF. A large number of redox chemistry processes may alter the agglomeration and distribution of CeNPs and relevant materials,^41^ which may eventually influence its properties including ·OH scavenging efficiency and SOD-like characteristics.^42^ Thus, characterizing CeNPs or their functionalized derivatives in more realistic environments will be beneficial and promising in follow-up studies.

## Conclusions

In this study, we compared the ability of CeNPs and CeNRs in scavenging hydroxyl radicals (·OH) under physiologically relevant conditions. We found that CeNPs and CeNRs exert high ·OH scavenging activity in both PBS and SLF. In SLF, the ·OH scavenging potential of CeNPs increased 4-fold as the loading increases from 0.1 to 10 mg mL^−1^. In the same loading range, CeNRs showed 5–50-fold higher ·OH scavenging potential than CeNPs, which may be attributable to the higher surface area and defect density of CeNRs. Furthermore, we found the scavenging activity of CeNPs is pH-dependent, exhibiting higher scavenging efficiency under lower pH condition. The observed ·OH scavenging efficiency of CeNPs and CeNRs in SLF took into account the effect of antioxidants at concentrations close to the epithelial lung fluid, reflecting the redox activity of CeNPs and CeNRs under more realistic *in vitro* conditions than previous studies. These findings are of critical importance for a better understanding of the relative ROS scavenging efficiency of CeNPs comparing to conventional antioxidants. Moreover, these results are also important for making accurate dose–response curves predicting the toxicity or antioxidant characteristics of CeNPs or their functionalized derivatives in biological and environmental processes.

## Conflicts of interest

There are no conflicts to declare.

## Supplementary Material

RA-009-C9RA00642G-s001
