# Supplementary material for: Antioxidant activity of cerium dioxide nanoparticles and nanorods in scavenging hydroxyl radicals
Source: RSC Adv. 2019 Apr 9;9(20):11077–81. doi: 10.1039/c9ra00642g (PMC9063017; doi:10.1039/c9ra00642g)
Supplement: RA-009-C9RA00642G-s001 [file RA-009-C9RA00642G-s001.pdf]

Electronic Supplementary Material (ESI) for RSC Advances.
This journal is © The Royal Society of Chemistry 2019

Supporting Information

Antioxidant activity of cerium dioxide nanoparticles and nanorods in scavenging hydroxyl radicals

Alexander Filippi,[a†] Fobang Liu,[a,b†] Jake Wilson,[a] Steven Lelieveld,[a] Karsten Korschelt,[c] Ting Wang,[d,a] Yueshe Wang,[d] Tobias Reich,[e] Ulrich Pöschl,[a] Wolfgang Tremel,[c] Haijie Tong[*a]

[a] Multiphase Chemistry Department, Max Planck Institute for Chemistry, Mainz, 55128, Germany

[b] School of Chemical and Biomolecular Engineering, Georgia Institute of Technology, Atlanta, Georgia 30332, USA

[c] Institute for Inorganic Chemistry and Analytical Chemistry, Johannes Gutenberg University Mainz, Mainz, 55128, Germany

[d] State Key Laboratory of Multiphase Flow in Power Engineering, Xi'an Jiaotong University, Xi'an 710049, China

[e] Institute of Nuclear Chemistry, Johannes Gutenberg University Mainz, Mainz, 55099, Germany

† Both authors contributed equally to this work

*Corresponding author:

Dr. Haijie Tong

E-mail: h.tong@mpic.de

Table of Contents

Section S1. Materials and solution samples..3

Section S2. Transmission electron microscope (TEM) analysis...4

Section S3. Brunauer-Emmett-Teller (BET) measurement...4

Section S4. XPS measurement 4

Section S5. CW-EPR measurement..7

Section S1. Materials and solution samples

The Ø 25 (544841) and Ø 50 nm (700290) diameter CeNPs were purchased from Sigma Aldrich and used as received without further purification. Homemade CeNRs were synthesized along the method described by Korschelt et al..[1] Briefly 0.08 M cerium (III) nitrate hexahydrate was mixed with 4.8 M sodium hydroxide for 30 min. Then, the reaction mixture was dried at 373 K for 24 h and cooled down to room temperature. Later on, the precipitates were isolated and washed with water and ethanol. Finally, the CeNRs were heated at 333 K for 24 h. These CeNRs have the advantages of low costs, environmental compatibility, and long-term stability,[1] exhibiting enzyme activity comparable to native jack bean urease[1] and quorum-sensing-regulatory properties similar to natural or engineered vanadium haloperoxidases and halogenases.[2] All the commercial CeNPs and homemade CeNRs were stored in closed vessels at room temperature until analysis.

The composition of surrogate lung fluid (SLF) is shown in Table S1. Therein the concentrations of ascorbate (11140, Sigma Aldrich), citric acid (C0759, Sigma Aldrich), glutathione (G4251, Sigma Aldrich), uric acid (U2625, Sigma Aldrich), and sodium chloride (443824T, VWR) are 0.2, 0.3, 0.1, 0.1, and 114 mM, respectively.[3] The pH was adjusted by using different molar ratios of sodium phosphate dibasic (71269, Fluka) and potassium phosphate monobasic (11594, Alfa Aesar). The total concentration of PO_4^{3-} is 10 mM. The pH values were measured using a S210 pH meter (Mettler Toledo). All the suspensions were always prepared and used freshly.

The loading of CeNPs in different suspensions is in the range of 0.1-30 mg mL^{-1}. The composition of different aqueous mixture samples is shown in Table S1. Briefly, the measured solution samples include:

1) Suspensions containing CeNPs (Ø 50 nm) and phosphate buffer (PBS).

2) Suspensions containing CeNPs (Ø 50 nm), PBS, and H_2O_2.

3) Suspensions containing CeNPs (Ø 50 nm), PBS, H_2O_2, and Fe^{2+}.

4) Suspensions containing CeNPs or CeNRs, antioxidants, PBS, H_2O_2, and Fe^{2+}.

Table S1. Summary of the spin trapping EPR experiments performed in this study.

Solutions	Component	Fenton reagents	Particle type	Effect on $OH^{\cdot}$ yield*	
				pH=4.7	pH=7.4
PBS	10 mM mixture of Na_2HPO_4 and KH_2PO_4	No	CeNPs (Ø 50 nm)	+	+
PBS		Only H_2O_2	CeNPs (Ø 50 nm)	++	+
PBS		H_2O_2 + Fe^{2+}	CeNPs (Ø 50 nm)	---	--
SLF	10 mM Na_2HPO_4 and KH_2PO_4<br>0.2 mM ascorbate<br>0.3 mM citric acid<br>0.1 mM glutathione<br>0.1 mM uric acid<br>114 mM NaCl	H_2O_2 + Fe^{2+}	CeNPs (Ø 50 nm)		-
SLF		H_2O_2 + Fe^{2+}	CeNPs (Ø 25 nm)		-
SLF		H_2O_2 + Fe^{2+}	CeNRs		---

* Note: the '+' and '-' represent the positive and negative effects of CeNPs or CeNRs in H_2O_2 formation, respectively. The number of the '+' and '-' indicates the extent of the effects by these NPs.

Section S2. Transmission electron microscope (TEM) analysis

The particle size and shape of the CeNPs and CeNRs were characterized using a TEM (FEI Tecnai G2 Spirit).[1] The TEM images of these nanoparticles were taken with an acceleration voltage of 120 kV and a LaB6 kathode.

Section S3. Brunauer-Emmett-Teller (BET) measurement

The surface area of CeNPs and CeNRs was determined through the BET method. A gas adsorption setup Autosorb-6B from Quantachrome with nitrogen as carrying gas has been used. The measurement was conducted at 77 K. Triplicates have been made for each type of nanoparticles.

Section S4. XPS measurement

X-ray photoelectron spectrometry (XPS) measurements were conducted to detect the surface composition of CeNPs and CeNRs. A XPS spectrometer (SPECS GmbH, Germany) equipped with a twin-anode X-ray source XR 50, a hemispherical energy analyzer PHOIBOS 100 and 5 channeltron detectors has been used for this study. The CeNPs or CeNRs particles were pressed into indium foil without any additional prior treatment. Non-monochromatized Al Kα radiation (1486.6 eV) was used to record the XPS spectra. The software CasaXPS (Casa Software Ltd., UK) was used for data evaluation. More information about the analysis steps can be found in a previous study.[4] XPS measurements where conducted with a pressure inside the analyzer chamber of about 10^{-8} mbar. The survey scan was recorded at a constant analyzer pass energy E_p = 50 eV. High-resolution spectra of Ce 3d and O 1s were recorded with E_p = 13 eV. Ten sweeps of each spectrum were averaged. Fifty sweeps in the region of the outer valence molecular orbitals (OVMO) were measured with E_p = 30 eV. Data analysis steps to produce the spectra in Fig. 1 and Fig. S2 included satellite subtraction from Al Kα radiation and Shirley background calculation. Since the spectra did not show any C 1s intensity, the binding energy of the Ce $3d_{5/2}$ peak of CeO_2 was set to 882.3 eV to correct for surface charging. The line shape for fitting the individual components was GL (60). The only constrain used in the fits of the Ce 3d spectra concerned the full-width-at-half-maximum (FWHM) of the individual peaks. In the Ce 3d spectra, that are composed of the $3d_{5/2}$ and $3d_{3/2}$ doublets with two associated satellite peaks each, the FWHM of the corresponding pairs of peaks were independently fitted. Peak areas and positions were adjustable parameters during all fits. The quantitative analysis of the O 1s components for the three different types of nanomaterials used in this study can be found in Table S3. Clearly the abundance of surface hydroxide is much higher in the nanorods than in the commercial CeNPs. The electron binding energies are display in Table S2.

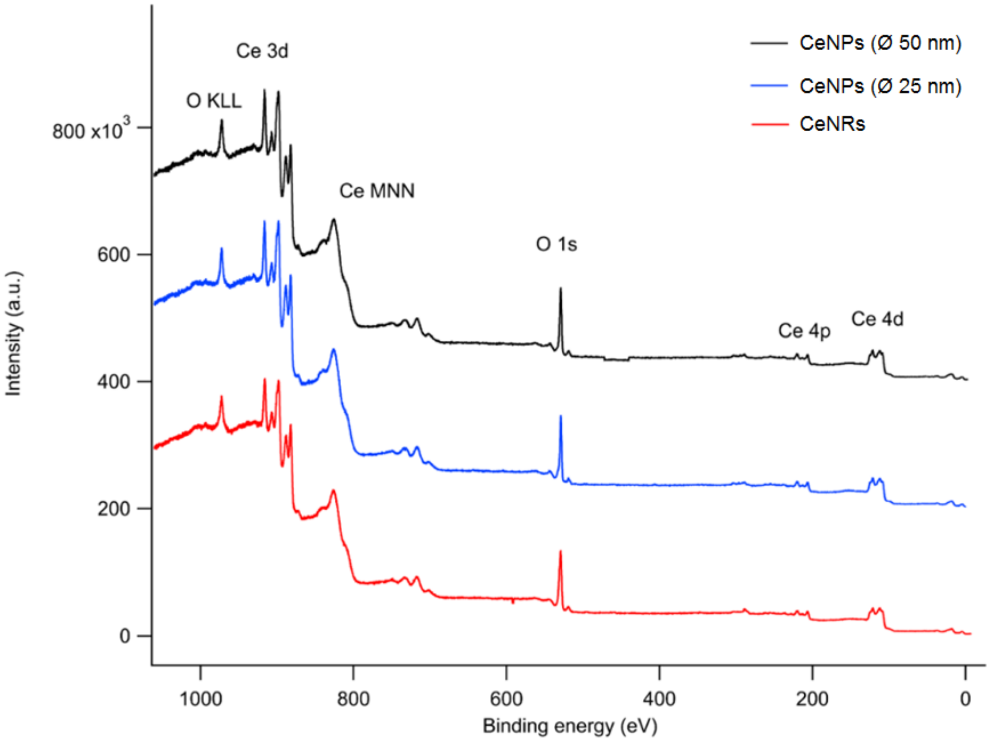

Fig. S1 XPS survey scan of the three different CeNPs and CeNRs samples.

Table S2. Electron binding energies E_b (eV) for all three types of CeO_2 nanoparticles. The FWHM is shown in parenthesis.

CeO_2	Ce $3d_{5/2}$*	Sat_1	Sat_2	O 1s (CeO_2)	O 1s (OH^-)
CeNPs (Ø 50 nm)	882.3 (2.5)	888.1 (5.8)	898.0 (2.3)	529.2 (1.4)	531.0 (1.9)
CeNPs (Ø 25 nm)	882.3 (2.4)	888.1 (5.7)	898.0 (2.3)	529.2 (1.4)	531.8 (2.8)
CeNRs	882.3 (2.6)	887.8 (6.0)	897.9 (2.5)	528.8 (1.5)	530.5 (2.5)

* E_b of Ce $3d_{5/2}$ in CeO_2 was set to 882.3 eV[4] to correct for electrostatic sample charging. The error in E_b equals ±0.1 eV. Sat_1 and Sat_2 are the charge-transfer and shake-up satellites associated to Ce $3d_{5/2}$, respectively.

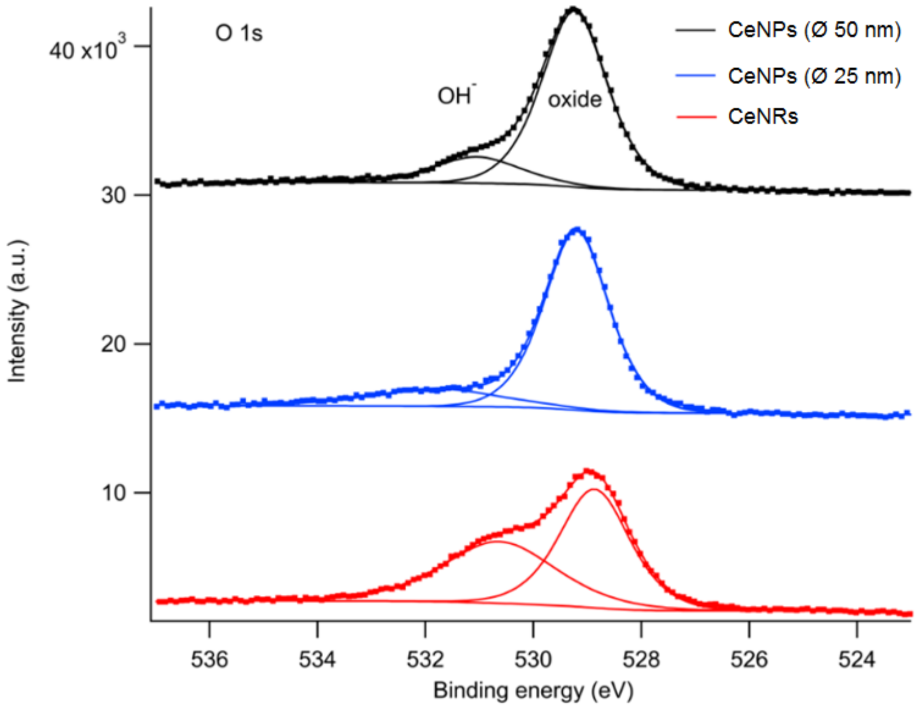

Fig. S2 XPS spectrum of the O 1s region of the three different nanoparticles. The CeNRs show significantly more OH^- groups[5] associated with the transition metal surface.

Table S3. Relative intensities (%) of the O 1s components and the oxygen coefficient *x* in CeO_{2-x} for different CeO_2 nanoparticles.

CeO_2	O 1s - CeO_2 (%)	O 1s - OH^- (%)	x^*
CeNPs (Ø 50 nm)	83	17	2.0
CeNPs (Ø 25 nm)	84	16	2.1
CeNRs	54	46	1.7

* The oxygen coefficient was calculated from the relative O 1s (oxide) and Ce $3d_{5/2}$ intensities, assuming that *x* equals 2.0 in sample CeNPs (Ø 50 nm). The relative error is approximately ±5%.

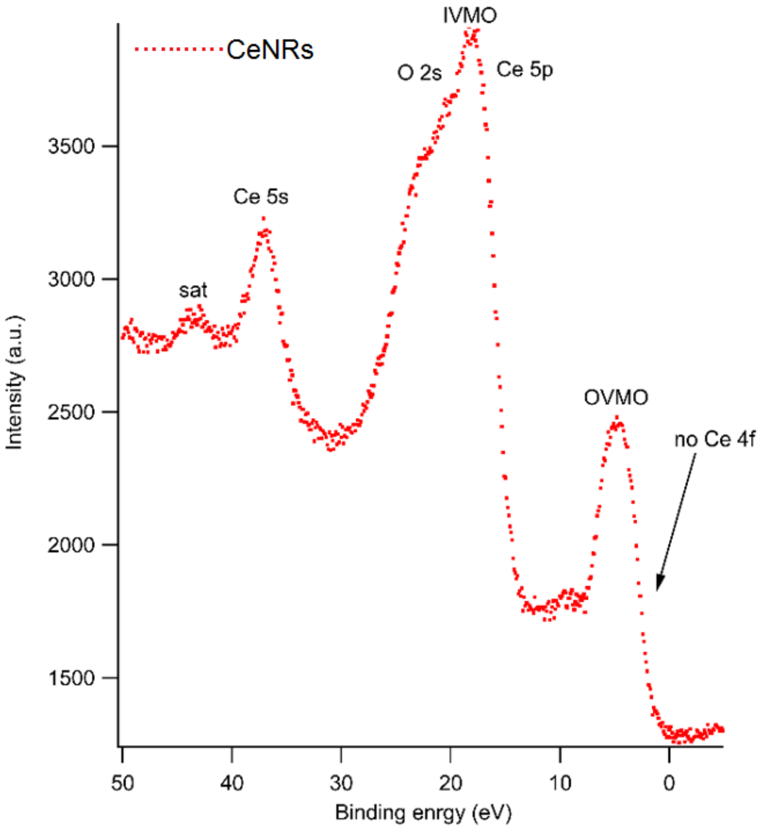

Fig. S3 Valence band XPS measurement of the CeNRs for the binding energy between 0 and 50 eV, displaying that there is no detectable Ce^{3+} abundance at the surface of the samples. In case of Ce^{3+}, this would have led to a shoulder at ~1.1 eV from unbound Ce 4f electrons as indicated by the arrow in the spectrum.

Section S5. CW-EPR measurement

In this study, 5-tert-butoxycarbonyl 5-methyl-1-pyrroline N-oxide (BMPO, high purity, Enzo Life Sciences GmbH) was used as trapping agent.[6] A continuous-wave electron paramagnetic resonance (CW-EPR) X-band spectrometer (EMXplus-10/12; Bruker Corporation) was used for measuring ·OH. Aqueous mixtures containing CeNPs and CeNRs were stirred with a vortex shaker (Heidolph Reax 1) for 10 min before EPR measurement. Then, approximately 20 µL of the suspension were immediately transferred into a micropipette for EPR analysis, which took <0.5 min. Thus, the reactants in each suspension sample totally reacted for 30±0.5 min at room temperature during our measurement. The concentration of BMPO in this study is 10 mM for all samples. The EPR spectra were recorded by setting the operating parameters as shown in Table S1. The spin-counting method embedded in the Bruker software, Xenon, was used to quantify BMPO-OH adducts. The spin-counting method was calibrated using the standard compound 4-hydroxy-2, 2, 6, 6-tetramethylpiperidin-1-oxyl (TEMPOL). EPR spectra were fitted and simulated using the Xenon software before quantification.[5]

Table S4. EPR settings used in this study.

Parameter	Value
Microwave frequency	9.84 Ghz
Microwave attenuation	20 dB
Microwave power	0.017 mW
Receiver gain	40 dB
Modulation amplitude	1 G
Scan number	50 scans
Center field	3509 G
Sweep width	100 G
Modulation frequency	100 kHz
Conversion time	11.05 ms
Time constant	10.24 ms

Table S5. BMPO-OH yield of aqueous mixtures of 1 mM $FeSO_4$, 10 mM H_2O_2, and varying loading of CeNPs (Ø 50 nm) in PBS with pH=4.7.

CeNP (Ø 50 nm) (mg/mL)	BMPO-OH (µM)
0	53.2±2.8
0.1±0.03	50.8±3.1
0.3±0.1	46.1±2.6
0.5±0.01	38.2±2.7
1.2±0.06	36.6±1.6
2.5±0.1	34.0±1.9
5.2±0.1	34.0±1.8
10.0±0.06	30.2±1.8
15.0±0.1	26.5±1.7
19.9±0.1	22.1±2.2
30.5±0.1	14.6±2.0

Table S6. BMPO-OH yield of aqueous mixtures of 1 mM $FeSO_4$, 10 mM H_2O_2, and varying loading of CeNPs (Ø 50 nm) in PBS with pH=7.4.

CeNP (Ø 50 nm) (mg/mL)	BMPO-OH (µM)
0	16.7±1.0
0.4±0.01	15.9±1.2
0.6±0.03	15.5±1.3
1.1±0.06	14.7±1.4
2.6±0.03	14.2±1.4
4.9±0.1	13.8±1.4
10.1±0.1	12.4±1.2
15.1±0.06	11.7±1.2
20.0±0.1	10.4±1.0
29.9±0.06	10.8±1.1

Table S7. BMPO-OH concentrations in aqueous mixtures of 1 mM $FeSO_4$, 10 mM H_2O_2, PBS, antioxidant, or SLF solutions with varying loadings of CeNPs or CeNRs.

PBS/Antioxidant/CeO_2	Fenton chemistry (µM)	Fenton+CeNPs (Ø 50 nm) (µM)	Fenton+CeNPs (Ø 25 nm) (µM)	Fenton+CeNRs (µM)
PBS	26.1±0.6			
SLF	16.4±2.6			
Citric acid	26.9±0.6			
Glutathione	24.0±7.1			
Uric Acid	19.9±7.2			
Ascorbate	15.8±2.9			
0.1		16.4±1.7	15.4±0.8	1.9±0.1
0.25		15.0±1.8	14.7±1.3	1.2±0.02
0.5		13.8±0.8	13.3±1.3	1.1±0.07
1.0		13.2±1.5	11.8±1.3	1.6±0.6
2.5		12.3±1.6	9.0±1.0	below LOD
5		10.3±1.1	1.2±1.1	below LOD
10		4.0±0.4	below LOD	below LOD
20		0.6±0.1	below LOD	

Notes and references

1. K. Korschelt, R. Schwidetzky, F. Pfitzner, J. Strugatchi, C. Schilling, M. von der Au, K. Kirchhoff, M. Panthofer, I. Lieberwirth, M. N. Tahir, C. Hess, B. Meermann and W. Tremel, *Nanoscale*, 2018, **10**, 13074-13082.
2. K. Herget, P. Hubach, S. Pusch, P. Deglmann, H. Gotz, T. E. Gorelik, I. A. Gural'skiy, F. Pfitzner, T. Link, S. Schenk, M. Panthofer, V. Ksenofontov, U. Kolb, T. Opatz, R. Andre and W. Tremel, *Adv. Mater.*, 2017, **29**, 1603823.
3. J. G. Charrier and C. Anastasio, *Atmos. Environ.*, 2011, **45**, 7555-7562.
4. K. I. Maslakov, Y. A. Teterin, A. J. Popel, A. Y. Teterin, K. E. Ivanov, S. N. Kalmykov, V. G. Petrov, P. K. Petrov and I. Farnan, *Appl. Surf. Sci.,* 2018, **448**, 154-162.
5. J. Haber, J. Stoch and L. Ungier, *J. Electron Spectrosc. Relat. Phenom.*, 1976, **9**, 459-467.
6. H. Tong, P. S. J. Lakey, A. M. Arangio, J. Socorro, F. Shen, K. Lucas, W. H. Brune, U. Poschl and M. Shiraiwa, *Environ. Sci. Technol.*, 2018, **52**, 11642-11651.
